# Acidogenic Potential of Plain Milk, Milk with Sugar, Milk with Cornflakes and Milk Cornflakes with Sugar: A Comparative Study

**DOI:** 10.5005/jp-journals-10005-1367

**Published:** 2016-09-27

**Authors:** Sham S Bhat, Sundeep K Hegde, Vidya S Bhat, KM Ramya, Praveen S Jodalli

**Affiliations:** 1 Head, Department of Pedodontics and Preventive Dentistry Yenepoya Dental College, Mangaluru, Karnataka, India; 2 Professor, Department of Pedodontics, Yenepoya Dental College Mangaluru, Karnataka, India; 3 Professor, Department of Prosthodontics, Yenepoya Dental College Mangaluru, Karnataka, India; 4 Assistant Professor, Department of Pedodontics and Preventive Dentistry, A.J Institute of Dental Science, Mangaluru, Karnataka, India; 5 Reader, Department of Public Health Dentistry, Yenepoya Dental College, Mangaluru, Karnataka, India

**Keywords:** Cornflakes, Milk, Salivary pH, Sugar.

## Abstract

**Aim:**

To compare the acidogenic potential of plain milk, milk with sugar, milk with cornflakes, and milk cornflakes with sugar by assessing the salivary pH.

**Materials and methods:**

The study was carried out on 40 school children of 8 to 12 years; 20 boys and 20 girls were randomly selected. The salivary pH was assessed before and after the consumption of milk; milk and sugar; milk and cornflakes; and milk, sugar, and cornflakes. Baseline unstimulated saliva was collected in sterile plastic tube and the pH was recorded. The change in the salivary pH from the respective groups after consuming the test meal was recorded as follows: (1) after 5 minutes; (2) after 10 minutes; (3) after 15 minutes; (4) after 30 minutes; (5) 120 minutes. Statistical analysis was done using Statistical Package for the Social Sciences 18.0 (SPSS).

**Results:**

The average baseline salivary pH among all the groups was 7.26. A fall in pH at 5 minutes was seen in all the four groups. However, at different time intervals 5, 10, 15, 30, and 120 minutes, the pH values between the groups showed a significant difference at p < 00.7, 0.005, 0.001, 0.010, and 0.028 respectively.

**Conclusion:**

The fall in pH in all the groups was not significant to a limit of critical pH. Milk when added with sugar and/or cornflakes as a meal did not pose a threat as there was not significant decrease in pH.

**How to cite this article:**

Bhat SS, Hegde SK, Bhat VS, Ramya KM, Jodalli PS. Acidogenic Potential of Plain Milk, Milk with Sugar, Milk with Cornflakes, and Milk Cornflakes with Sugar: A Comparative Study. Int J Clin Pediatr Dent 2016;9(3):218-221.

## INTRODUCTION

Nutrition for children is based on the same principles as nutrition for adults. Children, however, need different amount of specific nutrients, such as vitamins, minerals, carbohydrates, proteins, and fats at different ages. An appropriate diet is one that provide adequate nutrition and is appropriate for a child’s development.^1^

As the mankind has evolved, a drastic change has been seen in the dietary fermentable carbohydrate which eventually is associated with increased prevalence of dental caries. Milk is considered as an ideal food for the growing child. It is a more popular form of nutrition.^2^ Addition of a cereal to the diet is considered as healthy, and the most commonly used cereal is cornflakes and milk. The base ingredient of cornflakes is corn; sugar, malt flavoring agent, and high fructose corn syrup are the other major ingredients of cornflakes.

Cornflakes are often consumed with milk, sugar, and honey to add flavor. This eventually increases sugar content of the cereal, putting the children at a higher risk of dental caries.

In early 2014, World Health Organization (WHO) called for a dietary sugar intake restricted to 5% of total dietary calories in order to tackle public health problems, such as obesity and tooth decay.

The acidogenic potential of dietary products is well documented and there is sufficient evidence regarding the effect of saliva in controlling the plaque pH and that stimulation of saliva by foods is an important factor in determining their acidogenic potential.^3^ There is no substantial evidence regarding the acidogenic potential of milk when consumed with a cereal, i.e., cornflakes. This study was conducted to evaluate and compare the acidogenic potential of milk; milk and sugar; milk, cornflakes, and milk cornflakes with sugar at various time intervals.

## MATERIALS AND METHODS

The study was approved by the Ethics Committee of Yenepoya University, Mangaluru. A letter providing all the information and detail of the study was given to the parents and the consent was obtained.

### Inclusion Criteria

- School children aged between 8 and 12 years
- Decayed missing filled teeth (DMFT) index score should be less than or equal to 3
- Children who had consent from their parents.

### Exclusion Criteria

- Children who were physically challenged with gross orofacial defects like cleft lip and cleft palate
- Children who were medically compromised
- Children who were on antibiotics in the last 1 month.

*Selection of study subjects:* An initial oral screening of 158 children aged 8 to 12 years was done and DMFT was recorded. 40 children (Female:Male 20/20) who met the inclusion criteria were randomly selected. Study subjects were allocated into the following groups randomly:

*Group I:* Plain milk (250 mL)

*Group II:* Milk and sugar (250 mL + 1 tbl spoon sugar)

*Group III:* Milk and cornflakes (250 mL + 50 gm cornflakes)

*Group IV:* Milk, cornflakes, and sugar (250 mL + 50 gm cornflakes + 1 tbl spoon sugar).

The children were given a thorough oral prophylaxis 24 hours prior to the conduct of the study to ensure uniform baseline. On the day of study, the children were instructed not to eat any form of food or breakfast, except plain water. The study was conducted between 8 and 10 am.

*Saliva collection:* Baseline unstimulated saliva was collected into a dry, millimetric, sterile plastic tube, and the pH was recorded. After recording the resting pre consumption pH, the change in the salivary pH in the respective groups after consuming the test meal was recorded: (1) after 5 minutes; (2) after 10 minutes; (3) after 15 minutes; (4) after 30 minutes; (5) 120 minutes using a pH meter (Mettur Toledo MP220 pH meter; Schwarzenbach, Switzerland).

**Table 1:** **Table 1:** Mean pH at different time intervals

*I.D.*		*Baseline pH*		*5 minutes*		*10 minutes*		*15 minutes*		*30 minutes*		*120 minutes*	
1		7.18		7.03		6.99		7.04		7.16		7.28	
2		7.3		7.21		7.37		7.35		7.43		7.49	
3		7.21		6.79		6.93		6.97		6.97		7.16	
4		7.35		6.8		7.05		7.19		7.15		7.38	
Significance (p-value)		0.41		0.007*		0.005*		0.001*		0.01*		0.02*	

*Statistically significant values obtained using Kruskal-Wallis test

### Statistical Analysis

Data were analyzed using the SPSS 18 statistical program (SPSS, Inc., Chicago, YL, USA). Kruskal-Wallis test, Mann-Whitney test, and Wilcoxon signed rank test were used. p < 0.05 was accepted as significant.

## RESULTS

The average baseline salivary pH in all the groups was 7.26. Table 1 shows the mean pH at different time intervals among the groups. At baseline, there was no statistical significant difference in pH between the four groups. However, at different time intervals 5, 10, 15, 30, and 120 minutes, the pH values between the groups showed a significant difference (p < 00.7, 0.005, 0.001, 0.010, and 0.028 respectively).

**Graph 1:**
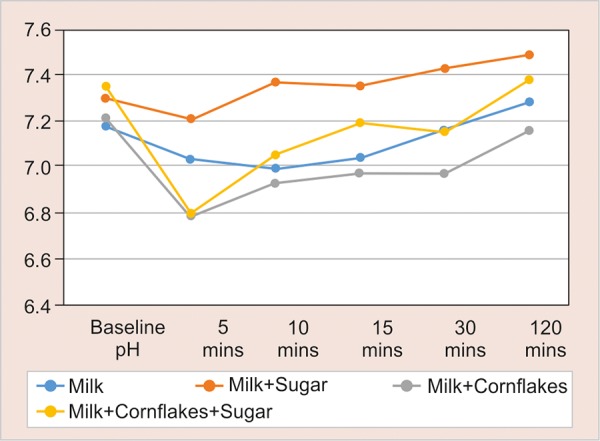
The pH at different time intervals in all the groups

Graph 1 explains the fall of pH at 5 minutes in all the four groups. The baseline pH in milk group (group I) was 7.18, a fall of pH at 5 to 7.03 minutes, 10 to 6.99 minutes, and 15 to 7.04 minutes, and a further rise of pH to baseline level was seen at 30 to 7.16 minutes and 120 to 7.28 minutes.

*Milk and sugar (group II):* The baseline pH decrease was 7.3. This group showed a decrease in pH at 7.21 at 5, 7.37 minutes at 10, 7.35 minutes at 15 minutes, and reached a baseline level at 30 to 7.43 minutes and 120 to 7.49 minutes.

*Milk and cornflakes (group III):* The baseline pH was 7.21. This group showed a significant fall in pH at 5 minutes (6.79), 10 minutes (6.93), 15 minutes (6.97), 30 minutes (6.97), and reached to baseline level only at 120 minutes (7.16).

*Milk, sugar, cornflakes (group IV):* The baseline pH was 7.35; a significant fall was seen at 5 minutes (6.8), 10 minutes (7.05), 15 minutes (7.19), and 120 minutes (7.38).

**Table 2:** **Table 2:** Between-groups comparison at different time intervals

*Individual groups*		*Baseline pH (p-value)*		*5 minutes*		*10 minutes*		*15 minutes*		*30 minutes*		*120 minutes*	
1 and 2		0.353		0.218		0.005*		0.000*		0.011*		0.052	
1 and 3		0.971		0.089		0.631		0.579		0.315		0.280	
1 and 4		0.218		0.052		0.684		0.043*		0.631		0.436	
2 and 3		0.353		0.015*		0.001*		0.000*		0.003*		0.004*	
2 and 4		0.579		0.002*		0.009*		0.143		0.023*		0.353	
3 and 4		0.218		0.579		0.436		0.052		0.280		0.075	

*Statistically significant values obtained using Mann-Whitney test

Table 2 shows the group comparisons of pH at different time intervals. Statistically significant difference was seen at 10, 15, 30 minutes (p < 0.05, 0.00, 0.01) between plain milk (group I), milk and sugar (group II). No statistical significant difference was obtained between groups I and III at any given time intervals. When groups I and IV were compared, statistically significant difference was seen only at 15 minutes (0.04). High statistical significant difference was seen at 5, 10, 15, 30, and 120 minutes between groups II and III. Also, statistical significant difference was at 5, 10, and 30 minutes (p < 0.002, 0.009, 0.023).

Table 3 shows the comparison of baseline pH with pH at different intervals within each group. The milk group showed a significant decrease in pH at 10 minutes. However, pH returned to normal at 15 minutes. Milk and sugar group showed no statistical difference at any time interval. Milk and cornflakes showed significant decrease in pH at 5 minutes (0.05), 10 minutes (0.018), 15 minutes (0.02); however, pH returned to baseline at 30 and 120 minutes. Milk, sugar, and cornflakes group show a significant decrease in pH at 5, 10, and 15 minutes (0.034); however, it returned to baseline at 120 minutes.

## DISCUSSION

Saliva plays a very important role in the maintenance of oral health. The ability of the saliva to buffer acids maintains the pH above critical levels, which thereby protects the teeth from demineralization.^4^ The average baseline pH of saliva of all the children in all the groups was 7.26. This finding is comparable to the baseline salivary pH seen in studies conducted by Anderson et al,^5^ Saigal and Tewari,^6^ and Schatele and Jenkins.^7^ The critical pH of saliva is 4.5 to 5.5.

The acidogenic potential of various groups is dependent on various contributing factors. Saliva has many essential functions. Salivary buffering capacity has been identified as one of the many factors that may affect an individual caries risk. The ability of saliva to buffer the acids is essential to maintain the pH above the critical pH, thus protecting teeth against demineralization.^8-10^

The consumption of milk; milk and sugar; milk and cornflakes; and milk, cornflakes, and sugar all lead to a fall in salivary pH, at 5 minutes, which further leads to decalcification.^11^ The result of the acidogenic challenge makes the tooth surface more prone to dental caries; hence this study was undertaken to assess and compare the acidogenic potential of the above four groups.

The mean baseline pH in the present study showed no significant differences with respect to gender in all the four groups. The presence of phosphate and bicarbonate ions in the saliva resists the effect on the enamel when the pH falls. The order of acidogenicity at 5 minutes after consumption was as follows: Milk cornflakes > Milk, sugar, cornflakes > Milk > Milk, sugar.

The pH drop in milk group to 6.99 at 5 minutes was seen, which recovered quickly at 15 minutes and reached baseline at 120 minutes. Milk has got a protective effect against dental caries even after adding sugar in the second group; here the decrease in pH was not significant at 10 minutes.

**Table 3:** **Table 3:** Comparison of baseline pH with pH at different time intervals within each group

		*p-value*									
*Group*		*Baseline-5 minutes*		*Baseline-10 minutes*		*Baseline-15 minutes*		*Baseline-30 minutes*		*Baseline-120 minutes*	
1		–0.15		–0.19		–0.14		–0.02		+0.1	
		0.082		0.015*		0.166		0.905		0.398	
2		–0.09		+0.07		+0.05		+0.13		+0.19	
		0.305		0.368		0.194		0.027*		0.007*	
3		–0.42		–0.28		–0.24		–0.24		–0.05	
		0.005*		0.013*		0.020*		0.106		0.608	
4		–0.55		–0.3		–0.16		–0.2		+0.03	
		0.005*		0.041*		0.090		0.034*		0.478	

– Decrease in pH; + Increase in pH; *Statistically significant obtained using Wilcoxon signed rank test

A significant pH drop was seen in milk cornflakes at 5, 10, 15, and 30 minutes. Then the pH returned to baseline at 120 minutes only, whereas in the milk, sugar, and cornflakes group the fall of pH was seen at 5 and 10 minutes only, then the pH returned to baseline at 30 and 120 minutes.

This study highlights the fact that though a similar pattern of pH drop at similar time intervals in all the groups has been seen, the recovery back to the baseline is prolonged only in the milk and cornflakes group and not when this was added with sugar, hence proving that the milk has got a protective effect against dental caries even after adding sugar. This concurs with the previous study done by Juneja and Kakade.^12^

Cornflakes, which are rich in dietary fibers, also showed significant caries protection. This was evident from the fact that difference between milk; cornflakes; and milk, sugar, and cornflakes was not statistically significant at any intervals of time. Hence, adding sugar did not decrease pH significantly, agreeing with earlier study by Pollard.^13^ To extrapolate the results of the study, the salivary pH immediate after the consumption should be evaluated with a larger sample size.

## CONCLUSION

The study concludes that milk when added with sugar and/or cornflakes as a meal did not pose a threat as there was no significant decrease of pH at a limit of critical pH. Hence, these alone or in combination can be recommended as a part of diet.
